# Paradigms of family medicine: bridging traditions with new concepts; meeting the challenge of being the good doctor from 2011

**DOI:** 10.1186/1447-056X-10-9

**Published:** 2011-07-16

**Authors:** John E Murtagh

**Affiliations:** 1 Emeritus Professor, Monash University, 1/270 Ferntree Gully Rd, Notting Hill, Victoria, 3168, Australia

**Keywords:** access to care, community medicine, continuity of care, Text, health promotion, international health, primary care, public health

## Abstract

This is the paper for the Wes Fabb Oration for the WONCA Asia Pacific Regional Conference 2011. This paper will review the case for the important role of the family physician/general practitioner in worldwide health care as determined by the WHO. The importance of continuing care is highlighted. The features of a good doctor will be defined and the process of meeting this challenge for excellence of care is presented.

## 

Family medicine/general practice remains the foundation stone of health service in the community. As the most interesting and challenging of medical disciplines it is based on six fundamental principles-primary care, family care, domiciliary care and continuing care which are all designed to achieve preventive and personal care [1]. In the contemporary climate where medical services are fragmented and there are competing interests there is a greater need than ever for the generalist. The patient whether sick or worried well requires a trustworthy consistent focal point and who better than the caring family doctor to take responsibility for the patient's welfare as trusted friend and advocate.

In 2008 the world health organisation (WHO) reaffirmed the importance of primary health care with its report "Primary health care now more than ever" [2]. The commentary emphasises that 'primary care brings promotion and prevention, cure and care together in a safe, effective and socially productive way at the interface between the population and the health system'. The key challenge is to "put people first since good care is about people" [2]. In 2009 the WHO Global Conference on Health Promotion resolved to reinvigorate primary health care, emphasising that effective health promotion is based on the renewal of this discipline [3]. To achieve this ideal the challenge for each of us is to be 'the good doctor' so that collectively we can make a significant difference.

An interesting survey on patient care by representative health consumers conducted at St Vincent's Hospital Melbourne revealed that the most important attributes of good doctors were (in some order of importance) caring, responsibility, empathy, interest, concern, competence, knowledge, confidence, sensitivity, perceptiveness, diligence, availability and manual skills. I do believe that we, as family physicians/general practitioners, deserve appropriate acknowledgement for outstanding service provided so often under difficulties. Pastor Peter Toon of the United Kingdom outlines his concept of a good general practitioner (GP) [4] in Figure 1. Dr Charles Boelen at the WHO proposed that a model efficient health care system will require family physicians/GPs who are "five star doctors" who can fulfil five essential functions [5] (Figure 2). The writer has proposed 10 guiding rules for a good GP based on clinical principles [6] (Figure 3).

**Figure 1:**
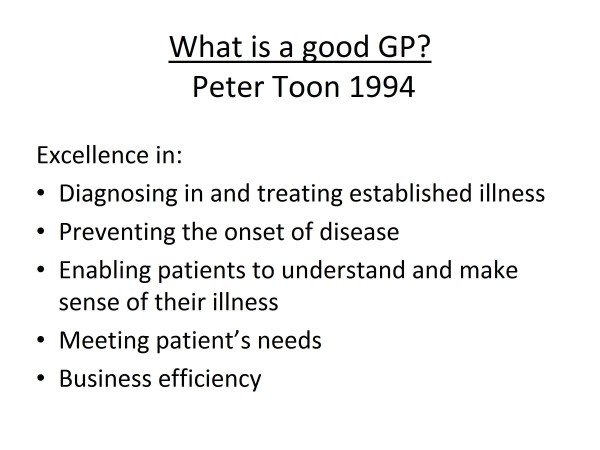
**What is a good GP? Peter Toon 1994**.

**Figure 2:**
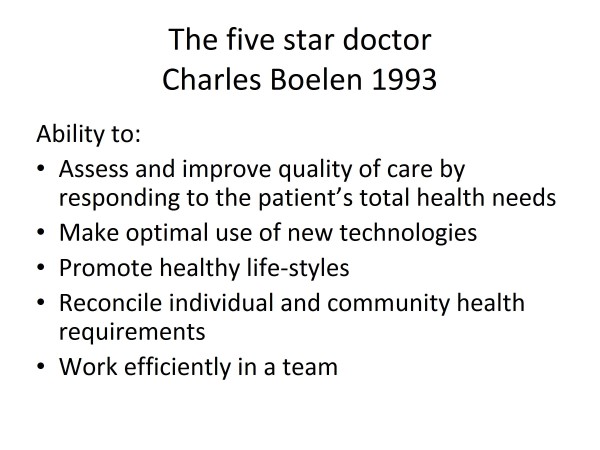
**The five star doctor**. Charles Boelen 1993.

**Figure 3:**
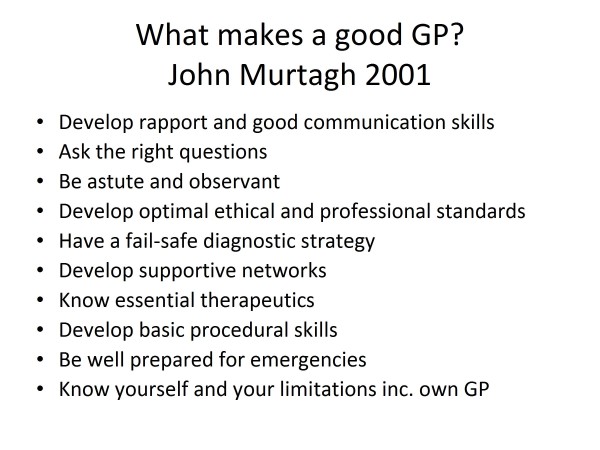
**What makes a good GP? John Murtagh 2001**.

The essence of family medicine is continuity of care and the evidence for its contribution to quality of care and better outcomes is presented in Figure 4[2]. Half a century in medicine has made me aware that there is a timeless and universal nature about disease and its management. People generally present with the same diseases, trauma, life events and mental illness. What changes is the method of management including diagnostic processes, delivery systems and professional development all of which are influenced by money which involves governance, services and technology.

**Figure 4:**
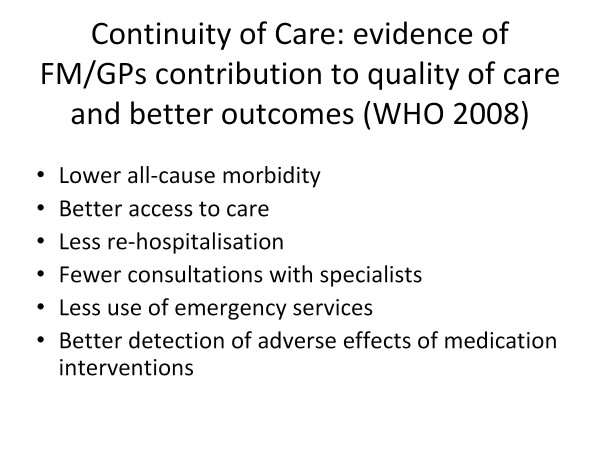
**Continuity of Care: evidence of FM/GPs contribution to quality of care and better outcomes (WHO 2008)**.

We can consider illness management broadly as acute and chronic care. In our modern developed countries the acute care especially of trauma and life threatening medical emergencies has been largely usurped by dedicated mobile emergency services that transport the patient to accident and emergency centres. While some of us may feel nostalgic about this situation we should accept that this is a positive situation for the community and the overworked family doctor. Nevertheless we still need to be prepared for and skilled at emergency management including cardiopulmonary resuscitation. We are visited every day by sick patients some of who can quickly develop into an emergency issue! Of course emergency care is the prime responsibility of the more isolated practitioner in rural and remote communities. These doctors who can manage every contingency are the real champions of our profession.

However our great area of responsibility and expertise is the management of chronic disease. The WHO has identified five priority millennium development goals namely diabetes mellitus, HIV/AIDS, tuberculosis, hypertension and mental health. The focus on addressing these diseases has been circumvented by the distraction of high morbidity infectious diseases such as malaria and the listed TB and HIV. A study by Piterman [7] of various international target conditions in chronic disease management has identified the 10 priority conditions listed in Figure 5. However the four lethal predisposing risk factors for arteriosclerotic disease are smoking, hypertension, hyperlipidaemia and diabetes mellitus- often referred to as 'the four horsemen of the apocalypse'.

**Figure 5:**
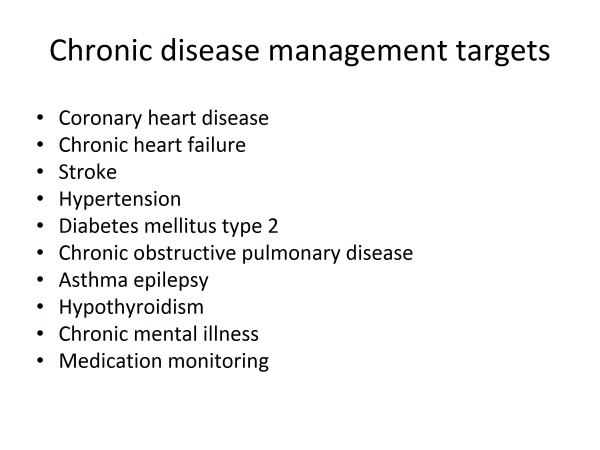
**Chronic disease management targets**.

Consideration of world's deadliest diseases (Figure 6) should help us focus on priorities on the world stage. The importance of astute diagnosis, rational use of antibiotics and preventive programs in particular becomes obvious. The inappropriate use of antibiotics worldwide and especially in the Asian sub-continent needs to be addressed by all practitioners.

**Figure 6:**
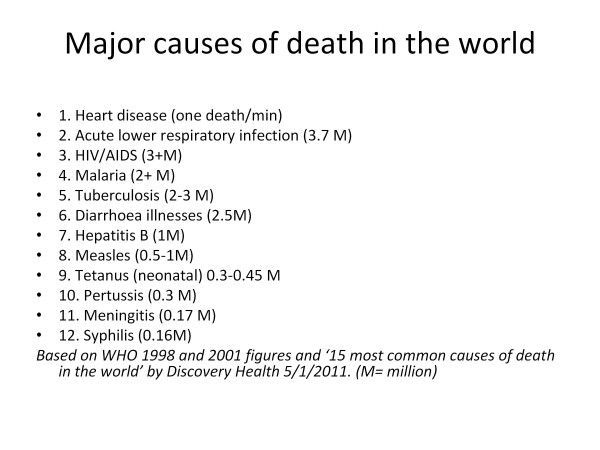
**Major causes of death in the world**. Based on WHO 1998 and 2001 figures and '15 most common causes of death in the world' by Discovery Health 5/1/2011. (M = million).

In the same spirit of concern for our environment we should be mindful of combating pollution and doing our small amount to influence a green and safer world. We do have a responsibility to keep abreast of the evidence base for climate change, global temperatures and carbon cycling. While in the Philippines it is interesting to ponder on the controversial assertion by Pilmer [8] that the eruption of Mt Pinatubo in 1991 led to the emission of more greenhouse gases into the atmosphere than the entire human race has emitted in its entire years on earth. There are 200 active volcanoes in the world. Toxic waste from industry as exemplified by the Hungarian toxic sludge spill last year is a critical issue and we should speak out against this issue as we should against war and nuclear weapons in particular.

Access to health services in remote areas and the developing world remains a problem and this includes the indigenous people of Australia. We must acknowledge the work of dedicated doctors, missionaries and organisations such as the United Nations, Red Cross and Medicins Sans Frontiers in particular for their extraordinary work under great hardship. The world continues to be burdened and challenged by man made disasters such as wars and natural disasters including the Haitian earthquake, drought, floods and tsunamis which overstretch the resources of the above mentioned health services. In many areas of the world there exists a workforce crisis and well trained allied health professionals are filling the vacuum. However in some countries including Australia our sovereignty as the focal point of primary health care is being challenged by some of these professionals particularly nurses and pharmacists. We must strive to be the experts through leadership and excellence of care.

In our own world of medical service we should continually strive to improve our basic knowledge including our diagnostic skills with one key objective being the early diagnosis of serious life threatening disease. One model that the writer has promoted for the past 35 years is that outlined in Figure 7[9] and this strategy forms the basis of analysing presenting symptoms in Murtagh's General Practice. One of the concerns facing all of us and especially working in countries where litigation for medical mistakes is a way of life is diagnostic delays, oversights or mistakes. We have to be particularly vigilant to detect serious disease especially infectious disease and the prime reason is for the benefit of our patient not so much facing medico-legal consequences.

**Figure 7:**
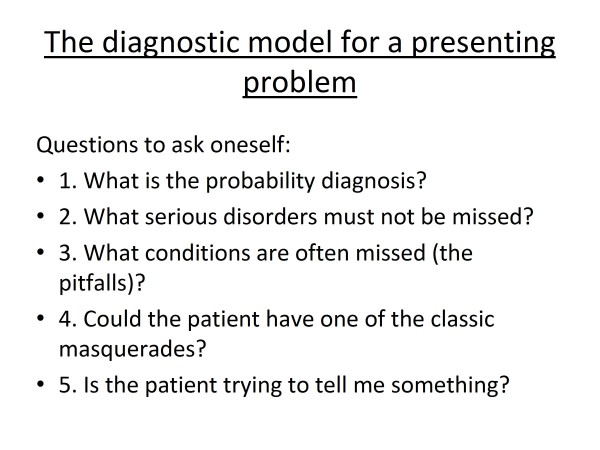
**The diagnostic model for a presenting problem**.

Another important paradigm to follow in our day to day practice is adherence where possible to scientific (as we called it in decades past) or evidence based medicine. This process applies more to tertiary based practice where huge decisions regarding interventions are necessary. However it does apply to our routine interventions and particularly prescribing in our practices. The WHO 2008 report emphasises the appropriate 'use of information and communication technologies to improve access, quality and efficiency in primary care [2]. The writer has made a small contribution to basic patient education (also known as doctor education) by the production of common patient handouts which are available for print out from GPs computers or for one page photocopying from the book 'Patient Education' [10].

The ever increasing influence of technology particularly computerisation of our practice presents a challenge to employ technology for productive ends. This includes the use of paperless records and e-Health. The WHO defines e-Health as 'the combined use of electronic communication and information technology in the health sector'. In more practical terms, 'it is the means of ensuring that the right health information is provided to the right person at the right place and time in a secure, electronic format for the purpose of optimising the quality and efficiency of health care delivery' [2].

An interesting yet concerning development in some countries is the rapidly changing ethical paradigms. The standards of 'utmost respect for human life' promoted by Hippocrates and the Declaration of Geneva have been eroded by the mindset of a burgeoning secular society. A survey of doctors in Australia revealed that the majority favoured euthanasia or physician assisted dying although one has to be careful in the interpretation of the questions posed in the surveys. One disturbing outcome of the 2008 Abortion Law Legislation in Victoria, Australia was the legalisation of abortion up unto late term without a conscience clause for GPs who are in fact obligated to facilitate a woman's choice for an abortion. The bill therefore changed the compliance with the human rights charter that the rights of conscience of medical practitioners should be respected. Interestingly the abortion lobby is linked with the legalise euthanasia lobby who have tried unsuccessfully 5 times in the past 2 years in Australia but I suspect that it will be eventually legalised as in some other countries.

Other areas of practice that should ideally be addressed by the well-rounded family physician/GP include teaching, research, health promotion through community education, collegiality and support for colleagues and professional development.

In conclusion it seems appropriate to paraphrase Dr Robert Rakel in his keynote presentation to the 14^th ^WONCA World Conference 'regardless of how computer literate we are or how high our technology or whether the setting is urban or rural, good medical care in the future will continue to depend on patient care provided by a concerned and compassionate family physician. The physician will be governed by ethics, not economics, by a partnership with the patient, not politics; and by compassion and communication, and not by capitation. Good medical care in the future will depend, as it does now and always has, on the quality of our interaction with the patient'[11].

## Competing interests

The author declares that he has no competing interests.
